# The Judicious Use of Stereotactic Radiosurgery and Hypofractionated Stereotactic Radiotherapy in the Management of Large Brain Metastases

**DOI:** 10.3390/cancers13010070

**Published:** 2020-12-29

**Authors:** Tyler Gutschenritter, Vyshak A. Venur, Stephanie E. Combs, Balamurugan Vellayappan, Anoop P. Patel, Matthew Foote, Kristin J. Redmond, Tony J. C. Wang, Arjun Sahgal, Samuel T. Chao, John H. Suh, Eric L. Chang, Richard G. Ellenbogen, Simon S. Lo

**Affiliations:** 1 Department of Radiation Oncology, University of Washington School of Medicine, Seattle, WA 98195, USA; tylergut@uw.edu; 2 Division of Medical Oncology, University of Washington School of Medicine, Seattle, WA 98195, USA; vyshakav@uw.edu; 3 Department of Radiation Oncology, Klinikum rechts der Isar, Technical University of Munich (TUM), 81675 Munich, Germany; Stephanie.combs@tum.de; 4 Institute for Radiation Medicine (IRM), Helmholtz Zentrum München, 85764 Neuherberg, Germany; 5 Department of Radiation Oncology, National University Cancer Institute, Singapore 119074, Singapore; Bala_vellayappan@nuhs.edu.sg; 6 Department of Neurological Surgery, University of Washington School of Medicine, Seattle, WA 98195, USA; apatel1@uw.edu (A.P.P.); rge@neurosurgery.washington.edu (R.G.E.); 7 Department of Radiation Oncology, Princess Alexandra Hospital, University of Queensland, ICON Cancer Care, Brisbane 4072, Australia; matthew.foote@health.qld.gov.au; 8 Department of Radiation Oncology and Molecular Radiation Sciences, The Johns Hopkins University, Baltimore, MD 21093, USA; kjanson3@jhmi.edu; 9 Department of Radiation Oncology, Columbia University Irving Medical Center, New York, NY 10032, USA; tjw2117@cumc.columbia.edu; 10 Department of Radiation Oncology, Odette Cancer Centre, Toronto, ON M4N 3M5, Canada; arjun.sahgal@sunnybrook.ca; 11 Department of Radiation Oncology, Cleveland Clinic, Cleveland, OH 44195, USA; chaos@ccf.org (S.T.C.); suhj@ccf.org (J.H.S.); 12 Department of Radiation Oncology, University of Southern California Keck School of Medicine, Los Angeles, CA 90033, USA; eric.chang@med.usc.edu

**Keywords:** stereotactic radiosurgery, hypofractionated stereotactic radiotherapy, large brain metastases, radionecrosis, local control

## Abstract

**Simple Summary:**

Brain metastases are the most common cause of cancerous brain tumors in adults. Large brain metastases are an especially difficult clinical scenario as patients often have debilitating symptoms from these tumors, and large tumors are more difficult to control with traditional single treatment radiation regimens alone or after surgery. Hypofractionated stereotactic radiotherapy is a novel way to deliver the higher doses of radiation to control large tumors either after surgery (most common), alone (common), or potentially before surgery (uncommon). Herein, we describe how delivering high doses over three or five treatments may improve tumor control and decrease complication rates compared to more traditional single treatment regimens for brain metastases larger than 2 cm in maximum dimension.

**Abstract:**

Brain metastases are the most common intracranial malignant tumor in adults and are a cause of significant morbidity and mortality for cancer patients. Large brain metastases, defined as tumors with a maximum dimension >2 cm, present a unique clinical challenge for the delivery of stereotactic radiosurgery (SRS) as patients often present with neurologic symptoms that require expeditious treatment that must also be balanced against the potential consequences of surgery and radiation therapy—namely, leptomeningeal disease (LMD) and radionecrosis (RN). Hypofractionated stereotactic radiotherapy (HSRT) and pre-operative SRS have emerged as novel treatment techniques to help improve local control rates and reduce rates of RN and LMD for this patient population commonly managed with post-operative SRS. Recent literature suggests that pre-operative SRS can potentially half the risk of LMD compared to post-operative SRS and that HSRT can improve risk of RN to less than 10% while improving local control when meeting the appropriate goals for biologically effective dose (BED) and dose-volume constraints. We recommend a 3- or 5-fraction regimen in lieu of SRS delivering 15 Gy or less for large metastases or resection cavities. We provide a table comparing the BED of commonly used SRS and HSRT regimens, and provide an algorithm to help guide the management of these challenging clinical scenarios.

## 1. Introduction

Brain metastases are the most common intracranial malignant tumor in adults and are a cause of significant morbidity and mortality for cancer patients [1,2]. The incidence of brain metastases in adults with solid tumors ranges between 10–30% and is dependent on histology and stage [3,4,5]. The histologies that account for the majority of brain metastases cases are non-small cell lung cancer (NSCLC), breast cancer, and melanoma [4]. Of patients with metastatic cancer at the time of initial diagnosis, an estimated 12% have brain metastases at diagnosis [1].

Patients with metastatic cancer, including those with brain metastases, are living longer due to recent improvements in systemic agents and radiation therapy [6,7,8]. However, the variability of central nervous system (CNS) penetration of novel systemic agents necessitates continued improvement of radiation therapy techniques for brain metastases. Current guidelines recommend definitive stereotactic radiosurgery (SRS) alone or in conjunction with surgical resection for patients with a limited number of brain metastases and good performance status [9,10]. SRS has largely supplanted definitive or post-operative whole brain radiation therapy (WBRT) for these patients after multiple studies demonstrated a decline in quality of life and neurocognitive function without providing a survival benefit [11,12,13,14,15,16].

Large brain metastases present a unique and common clinical challenge for the delivery of SRS. The published definition of “large” varies from >2 cm up to ≥4 cm in maximum diameter [17,18,19,20,21,22,23]. Patients often present with neurologic symptoms from vasogenic edema and mass effect, which makes definitive SRS a suboptimal treatment choice given the lack of immediate relief and risk of transient neurologic deterioration following treatment. Moreover, even post-operative SRS to a large resection cavity carries an increased risk of radionecrosis (RN) and may require dose reduction, potentially compromising local control [19,24,25,26,27,28,29,30,31,32].

Herein, we will review the recent developments in novel radiation and surgical treatment paradigms for managing large brain metastases and provide strategies for delivering SRS or hypofractionated stereotactic radiation therapy (HSRT) for a variety of clinical scenarios. For the purpose of this paper, we will define large brain metastases as >2 cm in maximal diameter but recognize that this definition is controversial and not universally accepted.

## 2. Optimal Approach for Utilizing Surgery and SRS

Post-operative radiation therapy prolongs overall survival in patients with a single brain metastasis and improves local control in patients with multiple brain metastases [11,15,33,34]. The 1-year local control rate following surgery without post-operative treatment is estimated to be 40–50% [15,33,34]. The 1-year local control rate with the addition of post-operative SRS is estimated to be 70–80% [11,15]. However, the 1-year local control rate for tumors >2 cm treated definitively or post-operatively with SRS leaves much room for improvement, with estimates ranging between 40% and 50% [15,19,30,31,32].

### 2.1. The Added Benefit of Resection to SRS

While post-operative radiation therapy has become the standard of care following resection of a brain metastasis, there is limited data comparing definitive SRS alone vs. surgical resection followed by SRS in patients with a limited number of brain metastases. Quigley et al. retrospectively examined local control and overall survival in 163 patients with 4 brain metastases or less who underwent either SRS alone (*n* = 113) or resection followed by post-operative SRS (*n* = 49) [35].

The resection plus SRS group had larger maximal tumor dimension (2.8 cm vs. 1.5 cm) and target volumes (9.9 mL vs. 3.6 mL), which resulted in lower average prescription dose (15.8 Gy vs. 17.5 Gy) compared to SRS alone. Gross total resection (GTR) was the only prognostic factor that significantly improved both local control and overall survival (*p* = 0.015 and *p* = 0.010). Furthermore, increased tumor volume was associated with significantly worse local control (*p* = 0.02), which is presumably related to the lower prescription doses delivered to these tumors or resection cavities. Overall, the results suggest that GTR plus post-operative SRS can provide excellent local control (22.5 months compared to 14.8 months for SRS alone) for patients with tumors >2 cm and can improve survival outcomes in select patients.

Prabhu et al. published a larger multi-institutional retrospective analysis examining local control and overall survival in 213 patients with 223 large brain metastases (>2 cm diameter) who either received SRS alone or GTR plus SRS [36]. There were 66 brain metastases in the SRS alone group and 157 brain metastases in the GTR plus SRS group. In contrast to Quigley et al., this study required GTR for inclusion, allowed for pre-operative SRS, and utilized competing risk analysis, which reduces overestimating rates of local recurrence when death is a competing risk.

Overall survival and local control were significantly improved in the GTR plus SRS arm. The SRS alone group had a median overall survival of 10 months compared to 15.2 months with GTR plus SRS (*p* = 0.01). The local failure rates at both 1 year and 2 years were significantly lower in the GTR plus SRS arm (1-year: 20.5% vs. 36.7%; 2-year: 25.6% vs. 43.1%; *p* = 0.007). There was no significant difference in overall survival, local control, and LMD when comparing pre-operative and post-operative SRS. However, the post-operative SRS group had significantly higher 1-year and 2-year rates of RN when compared to pre-operative SRS and SRS alone (1-year: 22.6% vs. 12.3% and 5%; 2-year: 33.5% vs. 17.2% and 9%; *p* < 0.001).

Taken together, the above studies both demonstrate that GTR plus SRS improves local control and overall survival in patients with brain metastases >2 cm, a limited number of brain metastases, and good performance status (ECOG 0–2). The average maximum tumor diameter for SRS alone groups was 1.5 cm in the Quigley et al. study and 2.25 cm in the Prabhu et al. study, but the tumor volumes in the resection plus SRS groups were similar (8.7 mL and 9.6 mL) between studies. Both delivered similar dose regimens for SRS alone (median dose: 17.5 and 18 Gy) and for resection plus SRS (15.8 and 15 Gy).

### 2.2. Pre-Operative versus Post-Operative SRS for Large Brain Metastases

Recently, much attention has been given to the optimal timing of SRS in the context of planned resection. This is a critical consideration in the treatment for large brain metastases that often present with neurologic symptoms, impending neurologic compromise, and/or significant vasogenic edema or mass effect—all of which are best managed with upfront surgical resection if possible. Post-operative SRS is currently considered the standard approach for all brain metastases, irrespective of size, when resection is required [11,15]. However, there are drawbacks to post-operative SRS, such as the following: (1) post-operative complications delaying initiation of radiation, especially with the more extensive resections required for large brain metastases; (2) the need for 1-mm or 2-mm expansion to the operative bed for optimal local control; and (3) the variable resection cavity dynamics between immediate post-operative MRI and SRS treatment planning MRI [37,38,39]. We have provided an overview of the advantages and disadvantages for SRS alone, post-operative SRS, and pre-operative SRS in Table 1 that we will discuss further in the following sections.

#### 2.2.1. Challenges of Post-Operative SRS for Large Brain Metastases

In regards to the data by Choi et al., this study retrospectively evaluated 120 cavities in 112 patients who received post-operative SRS and did not have prior intracranial radiation therapy. These results demonstrated significantly decreased 1-year local failure rates (3% vs. 16%; *p* = 0.042) without increased toxicity when placing uniform 2-mm expansion on the resection cavity compared to no expansion, but there was an increase in median planning target volume (PTV) by 55% with the 2-mm expansion [39]. However, likely due to the concern for an increased risk of RN with larger resection cavities, a 2-mm expansion has not been uniformly adopted for post-operative SRS. Specifically, a single institution, randomized controlled trial by Mahajan et al. examining post-operative SRS versus observation following surgical resection utilized only a 1-mm uniform expansion for PTV creation [15]. In contrast, a multi-institutional randomized controlled trial (N107C) examining post-operative SRS vs. post-operative whole brain radiation therapy (WBRT) utilized a uniform 2-mm expansion for PTV creation [11].

A recent retrospective analysis by Jhaveri et al. compared 1-mm expansion vs. >1-mm expansion (median 1.9 mm, mean 2.0 mm) for resected brain metastases in 133 patients with 139 cavities (1-mm group: 36 patients with 35 cavities; >1-mm group: 97 patients with 104 cavities) [38]. This study demonstrated that a 1-mm expansion had a similar 1-year local recurrence rate (15.2% vs. 14.3%) with a significantly lower risk of symptomatic RN compared to >1 mm expansion (1-year: 6% vs. 20.9%; 2-year: 9.1% vs. 26.6%; *p* = 0.028). Of note, HSRT was also associated with a significantly reduced risk of symptomatic RN (HR 0.13; *p* = 0.023).

The only factor associated with increased risk of local recurrence on multivariate analysis was a resection cavity volume >15 mL (HR 4.23; *p* = 0.047), which is an estimated 3.06-cm max dimension using spherical modeling. This study had a larger median resection cavity volume compared to the Choi et al. data (11.3 mL vs. 8.5 mL) and had slightly higher rates of local recurrence compared to Choi et al. (14–15% vs. 9%), likely due to the larger average cavity size. Overall, this study along with the data from Mahajan et al. suggests that a 1-mm expansion is safe and potentially has a lower risk of RN without compromising local control [15,38].

In regards to resection cavity volume dynamics, a retrospective study by Atalar et al. examining 68 cavities in 63 patients demonstrated that the median resection cavity volume was 29% smaller in volume compared to the pre-operative tumor volume (10.1 mL vs. 14.5 mL) [50]. However, the uniform expansion of 2 mm increased the median target volume to 15.6 mL and essentially negated the benefit of reduced target volume following resection [50]. In the 31 patients who had immediate post-operative and SRS planning MRI available for review in this study, there was no significant difference between cavity volumes, with approximately 4–5 weeks between imaging [50].

Nonetheless, there is conflicting data regarding the amount of resection cavity volume change between immediate post-operative imaging and pre-treatment SRS imaging. Contrary to the results from Atalar et al., a retrospective study by Jarvis et al. examining 43 cavities in 41 patients demonstrated that only 46.5% of resection cavities remained stable in size, with 23.3% decreasing in size by more than 2 mL and 30.2% increasing in size by more than 2 mL [51]. Similar findings were demonstrated in a retrospective analysis by Scharl et al., which examined 57 cavities in 57 patients found that the mean resection cavity volume decreased by 23.4% and decreased in 79.1% of cases examined [52]. Moreover, this study also found that the resection cavity continued to shrink between planning MRI and first follow-up MRI, with an average volume decrease of 20.7% [52].

Interestingly, the amount of T2 edema on immediate post-operative MRI has been demonstrated to be associated with a decrease in resection cavity volume on SRS planning imaging for cavities less than 4.0 cm in maximum diameter [53]. Of the 37 patients included in this study, 22 patients had resection cavities >2.0 cm in maximal diameter. The majority of patients (*n* = 24) had >2.0 cm maximal dimension of vasogenic edema (defined by T2 hyperintensity). This study determined that vasogenic edema measuring >1.5 cm was the factor that predicted a decrease in cavity size by 10% or more [53]. In sum, given the higher likelihood of large brain metastases presenting with significant vasogenic edema, the potential for cavity involution and the extent of post-operative edema potentially predicting this process are important concepts to consider when reviewing post-operative or treatment planning MRI and trying to determine SRS or HSRT for large resection cavities.

#### 2.2.2. The Potential Benefits of Pre-Operative SRS

The potential advantages of pre-operative SRS are as follows: (1) tumor is easy to identify and contour; (2) expansion margin is not required for target delineation uncertainty; (3) dose is often reduced by 20% given better oxygenation of intact tumor and planned surgery; and (4) there is potentially a reduction in leptomeningeal disease (LMD) risk following surgery [37]. When treating intact brain metastases with planned pre-operative SRS, the expansion margin is typically 0–1 mm with no utilization of volumetric expansion (i.e., GTV = PTV) being most commonly employed [37,49,54]. The lack of PTV margin and radiation dose reduction with planned pre-operative SRS reduces the volume of healthy brain tissue receiving 10–12 Gy, which are volumes that have been associated with increased RN [10,27,29,37,49,55,56]. Prabhu et al. reported a 1-year RN risk of approximately 5% and 2-year RN risk of 8.1–9% with pre-operative SRS delivered within 48 h of surgical resection [36,49]. Additionally, Patel et al. reported 2-year symptomatic RN rates of 4.9% for pre-operative SRS, which was significantly less than the 16.4% rate for post-operative SRS (*p* = 0.010) [45].

Regarding the potential decrease in risk of LMD, a multi-institutional retrospective study by Patel et al. found post-operative SRS to be associated with a significantly higher 2-year rate of LMD compared to pre-operative SRS (16.6% vs. 3.2%; *p* = 0.010) when examining 180 patients with 189 brain metastases who underwent resection and either pre-operative or post-operative SRS [45]. Furthermore, a separate multi-institutional retrospective study by Patel et al. found that patients receiving pre-operative SRS (66 patients with 71 lesions) had similar 2-year LMD rates (Pre-op SRS: 3.5% vs. WBRT: 9%) compared to patients receiving post-operative WBRT (36 patients with 42 cavities) [46].

Multiple studies have demonstrated that surgical resection of brain metastases is associated with higher rates of LMD compared to SRS alone [26,40,41,42]. These studies estimate the risk of LMD following SRS alone to be 5–6.2% [40,41,42]. Modern series examining rates of 1-year LMD with post-operative SRS estimate the rate to be 7.2–30% with the large heterogeneity potentially due to the variable definitions of LMD versus focal dural recurrence within the resection cavity [11,15,43,44,57]. LMD following resection is thought to be due to an increased risk of tumor spillage into the CSF at the time of surgery, which was not clinically relevant previously when post-operative WBRT was routinely used and potentially sterilized the intracranial CSF [46]. Additional risk factors for LMD post-operatively are breast cancer histology, piecemeal resection, posterior fossa location, multiple brain metastases, hemorrhagic features, and cystic features [43,45,58,59,60,61].

Another factor potentially contributing to the high rates of LMD with post-operative SRS is the use of a uniform 1–2-mm expansion without covering surgical tract or pre-operatively involved dura and venous sinus structures. This concern was addressed in the recent consensus contouring guidelines for post-operative SRS [62]. The authors recommended the CTV cover the entire resection cavity and surgical tract. Additionally, they recommended an additional 5–10-mm margin along the bone flap if the dura was involved pre-operative (1–5-mm margin if not involved pre-operatively) as well as a 1–5 mm margin along the venous sinus if the tumor abutted the sinus pre-operatively.

Reviewing these guidelines in the context of the two recently published clinical trials evaluating post-operative SRS against observation or post-operative WBRT, it should be noted that the 1-year rate of LMD for post-operative SRS was 7.2% in N107C trial compared to 28% in the Mahajan et al. study [11,15]. Of note, as discussed above, the N107C trial utilized a 2-mm PTV margin compared to the 1-mm PTV margin used by Mahajan et al., which suggests that even small margin modifications could potentially influence LMD rates in addition to RN rates. However, the risk of 1-year RN was <5% in both trials [11,15]. Unfortunately, large brain metastases often result in large resection cavities and additional coverage of the surgical tract and involved dura or venous structures results in a large target volume, which necessitates a lower prescription dose.

The N107C trial allowed the maximum resection cavity diameter to be up to 5 cm compared to Mahajan et al. excluding resection cavities with diameter >4 cm and having a median cavity volume of 8.9 mL (median volume not listed for N107C) [11,15]. The lower than expected 1-year local control rate of the N107C trial (61.8% vs. expected 70–80%) could be attributed to the uniform 2-mm expansion with lack of surgical tract coverage or prescription doses with too low of biologically effective doses for large resection cavities (17 Gy for 8.0–14.3 mL; 15 Gy for 14.4–19.9 mL; 12 Gy for 30 mL or more) [11]. However, the data by Mahajan et al. demonstrates a 1-year local control rate of 72% utilizing only a 1-mm expansion, but the 1-year local control rate drops to 40–46% for resection cavities >2.5 cm in maximum diameter (8.4 mL using spherical modeling) [15]. The authors of the N107C trial also comment that the local control rate could have been lower than expected due to SRS post-treatment change being falsely called recurrence [11].

Ultimately, pre-operative SRS provides a unique opportunity to provide equivalent local control, or possibly improved control for large tumors, with potentially decreased risk of RN and LMD due to the ability to reduce the dose by approximately 20%, sterilize the surgical bed pre-operative, and utilize little-to-no expansion. However, when considering pre-operative SRS or HSRT, it should be noted that the majority of the available data comes from a small group of authors with a shared database of prospectively and retrospectively obtained end points. These results may not be widely generalizable and may better reflect the specific results of the authors’ community and academic practices. Nonetheless, the preliminary results are promising and will hopefully spur further clinical research on this topic.

#### 2.2.3. The Pitfalls of Pre-Operative SRS and Advantage of Post-Operative SRS

The main pitfalls of pre-operative SRS and advantages of post-operative SRS are in the circumstances of benign surgical pathology, subtotal resection, and neurologic symptoms at presentation [37]. In the cases of benign surgical pathology, the patient could be spared from unnecessary radiation should the post-operative SRS paradigm be employed. In the initial study by Patchell et al. examining the benefit of surgery compared to WBRT, a total of 6 patients (11%) from the initially recruited cohort of 54 patients were excluded because final surgical pathology was benign [63]. Improvements in MRI and functional imaging have undoubtedly improved our ability to determine recurrent or metastatic disease from benign lesions, but the rate of false detection will likely never be 0%.

In the event of a subtotal resection, post-operative radiation therapy allows the opportunity to treat the residual disease definitively with an adequate dose. For cases of subtotal resection following pre-operative SRS, the scenario is complicated by the commonly utilized 20% dose reduction owing to the theoretical improved oxygenation of intact metastases. While the reduced dose regimen delivered pre-operatively is considered sub-therapeutic for definitive treatment, the current consensus recommendation is for surveillance and consideration of re-irradiation as a last resort for continued progression [37]. Nonetheless, cases of subtotal resection are difficult clinical scenarios with worse outcomes compared to GTR regardless of whether pre-operative or post-operative SRS is chosen [35,57].

Finally, it is not uncommon for patients with large brain metastases to experience mass effect and edema. In such situation, immediate relief of mass effect is necessary and preoperative SRS may not be suitable. Immediate surgical resection to relieve the mass effect followed by postoperative SRS is the more appropriate treatment.

## 3. The Appropriate Use of Hypofractionated Stereotactic Radiation Therapy (HSRT)

As discussed above, the control rate for large brain metastases is poor, with 1-year and 2-year rates between 40% and 50% with post-operative SRS [15,19,30,31,32,64]. Moreover, the volume of healthy brain tissue receiving 12 Gy (V12 Gy) has been correlated with risk of RN, especially for volumes >10 mL, with RN risks ranging between 15% and 55% [24,25,27,65,66]. HSRT provides the ability to improve local control and reduce the risk of RN by taking advantage of the manipulation of the biologically effective dose (BED) that fractionation can have on early and late responding tissues as described in the linear-quadratic model [67]. Table 2 demonstrates this effect on BED for the commonly employed fractionation schemes in SRS and HSRT.

Interest has increased for the utilization of HSRT, and multiple retrospective studies have been published examining the rates of local control and RN for HSRT [26,28,57,68,69,70,71,72]. The 1-year local control rate for HSRT following resection of a large brain metastasis ranges widely between 65% and 95% [26,57,68,69,70,71,72]. The rates of RN with HSRT range from 5% to 25% for the same cohort of patients with resected large brain metastases [26,57,72]. Recently published guidelines updating the radiation dose-volume tolerances of brain tissue for novel 3-fraction HSRT regimens reported the volume of normal brain tissue receiving 18 Gy (V18 Gy) as less than 30 mL and the volume of normal brain tissue receiving 23 Gy (V23 Gy) as less than 7 mL kept RN risk below 10% on a pooled analysis of available studies [65].

In regards to the optimal resection cavity volume or tumor diameter to begin considering HSRT over SRS, there is variability in the literature reporting the volume (or maximum diameter) cutoff where HSRT is superior in terms of local control or RN. From an early report by Schlienger et al., HSRT is preferable to SRS for tumors >2 cm in maximum diameter due to superior local control and decreased RN risk [73]. Multiple other studies suggest the decrease in local control becomes significant for SRS at pre-operative maximum tumor diameter >3 cm or resection cavity volume >15 mL [15,38,74].

A meta-analysis by Akanda et al. examined the impact of dose, fractionation, treatment volume margin, and time to SRS on 1-year local control for post-operative SRS compared to post-operative HSRT [75]. However, this analysis did not examine the effect of resection cavity size. The mean 1-year local control rate was 80% across the 24 studies that utilized post-operative SRS. There was no significant difference in mean 1-year local control when examining median SRS dose <18 Gy vs. ≥18 Gy (80% vs. 81%). In the 14 studies that utilized post-operative HSRT, the mean 1-year local control was 87.3%, which was significantly better than post-operative SRS (*p* = 0.021). There was no significant difference in mean 1-year local control when comparing the 21 studies that utilized an expansion margin compared to the 5 studies that did not employ an expansion on the resection cavity. The overall rate of RN was 6.9% for the 36 studies that reported this outcome. The overall rate of LMD was 12.6% for the 22 studies that reported this outcome.

A meta-analysis by Lehrer et al. that specifically looked at brain metastases >2 cm demonstrated a significantly reduced risk of RN for HSRT compared to SRS for tumors 2–3 cm maximum diameter with a median cavity volume of 9.8 mL (7.3% vs. 23.1%; *p* = 0.003) and provided a similar rate of 1-year local control (HSRT: 92.9% vs. SRS: 77.1%; *p* = 0.18) [26]. However, for tumors >3 cm (median resection cavity volume of 17.5 mL), there was no significant benefit for HSRT in the definitive or post-operative setting in terms of RN [26]. They did note a trend towards significance in the post-operative setting benefitting HSRT compared to SRS for 1-year local control in tumors >3 cm (HSRT: 85.7% vs. SRS: 62.4%; *p* = 0.13) with similar rates of RN (7.3% vs. 7.5%) [26].

In comparing these results to those of the meta-analysis by Akanda et al., the 1-year local control for HSRT in the 2–3-cm group and >3-cm group are similar to the mean 1-year local control rate of 87%. However, the 1-year local control for SRS in the >3-cm group is noticeably less than the mean 1-year local control rate of 80% demonstrated by Akanda et al. This discrepancy, the lack of dose effect on mean 1-year local control in the data from Akanda et al., and the similar 1-year local control rates between the HSRT groups suggests that the SRS trials included in the Akanda et al. analysis likely treated smaller resection cavities while the HSRT trials preferentially included larger resection cavities.

More recently, a single institution prospective registry study by Faruqi et al. examined asymptomatic and symptomatic RN in 187 patients with 118 surgical cavities and 132 intact metastases who were treated with 5-fraction HSRT using a 2-mm PTV expansion [76]. The median dose was 30 Gy (range: 20–35 Gy), and the median follow up was 12 months. The median resection cavity volume was 12.7 mL (maximum dimension 2.9 cm using a spherical model), and the median intact metastasis 2.9 mL (maximum dimension 1.8 cm using a spherical model). The median resection cavity PTV was 24.9 mL, and the median intact metastasis PTV was 7.7 mL. Overall, 64% of intact metastases or cavities were >2 cm in maximum dimension.

The RN rates in this patient cohort were 21.2% for asymptomatic RN and 10.8% for symptomatic RN [76]. The median time to asymptomatic RN was 7.9 months and 7 months for symptomatic RN. The 1-year RN risk for resection cavities was 12% compared to the 1-year RN risk of 22% for intact metastases. Multivariate analysis revealed significantly increased risk of RN for intact metastases compared to resection cavities (OR 3.5; *p* = 0.001) and for single metastasis treatment plans compared to multiple metastases treated in the plan (OR 3.7; *p* = 0.001). The significant risk factors that predicted increased risk for symptomatic RN were prior SRS (OR 8.7; *p* = 0.037) or WBRT (OR 7.7; *p* = 0.009) to the target. For resection cavities, only targeted immunotherapies within 1 month of treatment significantly increased the risk of RN on multivariate analysis (OR 17.4; *p* = 0.018).

Overall, this study demonstrated a 1-year local control of 84% for resection cavities and 78.2% for intact metastases [76]. Moreover, they demonstrated that a healthy brain volume minus the intact metastasis volume receiving 30 Gy (BMC30) was a significant risk factor for symptomatic RN with a threshold of 10.5 mL or more (OR 7.2; *p* = 0.02). The 1-year symptomatic RN rate was 13% for BMC30 <10.5 mL and 61% for BMC30 of 10.5 mL or greater. Ultimately, they believe the higher rate of RN in the intact metastasis group was related to significantly increased exposure to prior WBRT (32% vs. 13%; *p* < 0.001) and targeted immunotherapies (13% vs. 3%; *p* < 0.01) compared to the resection cavity cohort. They do not note any difference in radiation dose delivered between the intact metastases and resection cavities arms. However, they do recommend dose reduction from 30 to 27.5 Gy for intact metastases with BMC30 > 10 mL.

Lastly, a multi-institutional retrospective analysis by Eitz et al. examining outcomes and prognostic factors for patients receiving post-operative HSRT was recently published and provides additional insight to what was learned from the Lehrer et al. meta-analysis. This large multi-institutional analysis of 558 patients with 581 resection cavities demonstrated 1-year local control of 84% for a median dose of 30 Gy over 5 fractions with a median resection cavity volume of 10.9 mL (max diameter >2.6 cm using spherical modeling) and median PTV volume of 23.9 mL [57]. The 1-year rate of RN was 8.6% and 1-year rate of LMD was 13.1% [57]. On multivariate analysis, there was no significant difference in local control between PTV volumes <23 mL or those 23 mL and greater [57]. There was durable 2-year and 3-year local control rates of 75% and 71%, respectively, and patients with a controlled primary tumor (HR 0.59; *p* = 0.02) and a single brain metastases (HR 0.57; *p* = 0.03) had significantly improved local control on multivariate analysis. Lastly, multivariate analysis noted a significant detriment on overall survival when HSRT was delivered 22–33 days after resection compared to 0–21 days after resection (HR 1.50; *p* = 0.02), but there was no significant difference in local control when comparing these two groups [57].

The similar 1-year local control results between the Lehrer et al. (85.7–92.9%), Akanda et al. (87.3%), Faruqi et al. (84%), and Eitz et al. (84%) are very promising for HSRT despite varied fractionation schemes and median resection cavity volumes (9.8–18.5 mL vs. unknown vs. 12.7 mL vs. 10.9 mL). Multiple trials have examined the optimal dose fractionation scheme for HSRT in patients with large brain metastases [76,77,78,79,80]. A BED Gy_10_ of 50 Gy or more appears to provide significantly improved local control [78]. However, lower BED regimens have been demonstrated to provide similar control.

Specifically, a retrospective study by Keller et al. examined 181 patients with 189 resection cavities who received 23.1 Gy over 3 fractions to PTV prescribed to the 70% isodose line [81]. A 2-mm expansion was used to create the PTV, and the BED Gy_10_ of this regimen is 40.9 Gy. The 1-year local control was 88.2%, and the 1-year RN risk was 5.4%. While 54% of the 189 lesion were ≥3 cm pre-operatively, the median treated resection cavity volume was 7.6 mL. This volume is noticeably smaller than the volumes for the meta-analysis and retrospective studies listed above, which could explain the good outcomes with a lower BED Gy_10_ regimen.

As such, when choosing 3- or 5-fraction regimens to meet the recommended BED goal, the radiation oncologist must not only consider the resection cavity size but also consider the volume of healthy brain irradiated as well as the suggested brainstem and optic apparatus constraints. As mentioned above, limiting the healthy brain exposure to the following constraints has been shown to keep RN risk reasonably low: V18 < 30 mL and V23 < 7 mL for 3-fraction HSRT regimens as well as BMC 30 < 10.5 mL for 5-fraction regimens [65,76]. Additionally, the suggested maximum dose to the optic apparatus is 21–25 Gy in 5 fractions and 15–18 Gy over 3 fractions [79,80,82]. The suggested maximum dose to the brainstem is 31 Gy in 5 fractions and 23 Gy over 3 fractions [79,80,82]. To best balance these constraint goals with the above-mentioned BED goal, we have chosen 27 Gy over 3 fractions and 30 Gy over 5 fractions as the regimens with the best risk-to-benefit ratio and with the most robust amount of data to support their use. Furthermore, we would discourage the use of SRS to 15 Gy or less for large tumors or resection cavities and would instead recommend the above mentioned 3- or 5-fraction regimens.

For determining the best measurement to appraise when considering SRS or HRST, volumetric measurements of tumors or resection cavities are likely more valuable than maximum dimension measurements given the often non-spherical and variable shapes of resection cavities or intact metastases. Furthermore, recent and ongoing trials, namely N107C and A071801, have utilized volumetric measurements to guide prescription doses. Lastly, radiation dose constraints are often volumetric, and as discussed above, volumetric constraints for healthy brain predicting RN risk in the context of HSRT have recently been proposed for 3-fraction regimens (V18 < 30 mL and V23 < 7 mL) as well as for 5-fraction regimens (BMC30 < 10.5 mL) [65,76].

**Figure 1:**
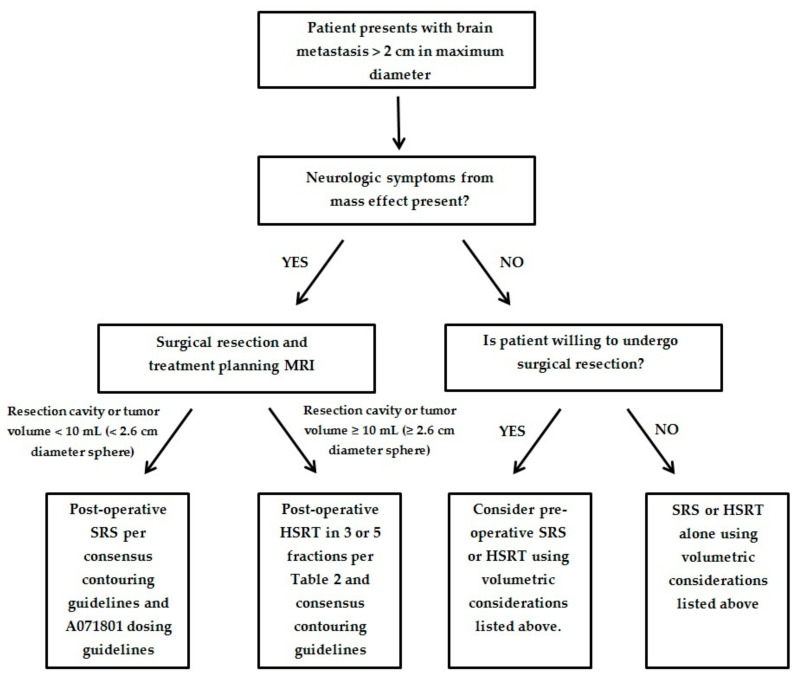
Proposed algorithm for management of patients with large brain metastasis.

We have provided an algorithm to help guide the optimal treatment of patients presenting with a large brain metastasis and the additional following criteria: (1) no more than 4 brain metastases; (2) a KPS of 60% or greater; (3) unresected metastasis <4.0 cm in maximum dimension; (4) resection cavity <5.0 cm in maximum dimension; and (5) the patient is medically fit for surgical resection (Figure 1). These criteria are similar to the current inclusion criteria for the ongoing Alliance A071801 trial examining local control in SRS compared to HSRT for large brain metastases. Until the results of the A071801 trial are available, we will have to rely on expert consensus and guidance utilizing the currently existing data outlined above. For patients unable to undergo surgery and who have 5–10 brain metastases with cumulative tumor volume <7 mL, we recommend SRS or HSRT alone per current consensus guidelines [10,83].

Lastly, in regard to the optimal duration between resection and SRS or HSRT, the A071801 protocol requires gross total resection and post-operative brain MRI within 30 days of pre-registration. The meta-analysis by Akanda et al. noted similar mean 1-year local control values for SRS within 30 days of resection or after 30 days of resection (85.1% vs. 82.7%) [75]. Moreover, the large retrospective study by Eitz et al. did not find a significant difference in local control on multivariate analysis when comparing 22–33 days between resection and HSRT to 0–21 days or ≥34 days [57]. The significantly decreased OS in the 22–33-day group could have been related to patients with post-operative complications or poor performance status recovering more slowly from surgery and experiencing worse survival independent of treatment timing. As such, we recommend post-operative brain MRI 3 to 4 weeks following resection to allow for optimal recovery and potential decrease in resection cavity size. Post-operative SRS or HSRT should occur within days of post-operative brain MRI.

## 4. Conclusions

The management of large brain metastases often presents a unique clinical challenge for oncologists to provide treatment with the best opportunity for disease control while limiting the long-term toxicity from treatment. These patients often present with neurologic symptoms that require expeditious treatment that must also be balanced against the potential consequences of surgery and radiation therapy—namely, leptomeningeal dissemination and radionecrosis. Early data suggests that HSRT and pre-operative SRS or HSRT are promising techniques to improve local control and minimize toxicity for patients with large brain metastases. We look forward to the results of A071801 and other future prospective randomized controlled trials examining these techniques. We recommend 3- or 5-fraction regimens in lieu of SRS delivering 15 Gy or less. We have provided a comparative dose-fractionation table and a management algorithm based on the current data to help guide treatment until more information is available.

## Figures and Tables

**Table 1:** Overview of the comparative advantages and disadvantages of SRS alone, post-operative SRS, and pre-operative SRS.

Parameter	SRS Alone	Post-Op SRS	Pre-Op SRS
Delay of systemic therapy	Minimal	Significant	Significant
Relief of neurologic symptoms	Risk of worsening	Immediate	Risk of worsening
CTV Margin	None	1–3 mm	None
Overall Survival	Worse	Better	Better
Local Control	Worse	Better	Better
1-year LMD Risk	5–7% [40,41,42]	7–30% [11,15,43,44]	≤5% [45,46]
1-year RN Risk	5–20% [36,47,48]	3–25% [11,36,38,39]	5–10% [36,45,49]
Prescribed Dose	Per RTOG 9005	Per N107C or A071801	20% Reduced
Timing	Within days	2–6 weeks post-op	48 h pre-op

**Table 2:** Commonly employed SRS and HSRT schemes and their associated BED for early and late responding tissues. BED Gy_10_ represents the BED for early responding tissue such as tumor cells. BED Gy_2_ represents the BED for late responding tissue, such as healthy brain cells. Gy = Gray, fx = fraction(s).

Regimen	BED Gy_10_	BED Gy_2_
30 Gy/5 fx	48.0	120.0
27 Gy/3 fx	51.3	148.5
18 Gy/1 fx	50.4	180.0
16 Gy/1 fx	41.6	144.0
14 Gy/1 fx	33.6	112.0
12 Gy/1 fx	26.4	84.0
